# Citizen Science Reveals Unexpected Continental-Scale Evolutionary Change in a Model Organism

**DOI:** 10.1371/journal.pone.0018927

**Published:** 2011-04-27

**Authors:** Jonathan Silvertown, Laurence Cook, Robert Cameron, Mike Dodd, Kevin McConway, Jenny Worthington, Peter Skelton, Christian Anton, Oliver Bossdorf, Bruno Baur, Menno Schilthuizen, Benoît Fontaine, Helmut Sattmann, Giorgio Bertorelle, Maria Correia, Cristina Oliveira, Beata Pokryszko, Małgorzata Ożgo, Arturs Stalažs, Eoin Gill, Üllar Rammul, Péter Sólymos, Zoltan Féher, Xavier Juan

**Affiliations:** 1 Department of Life Sciences, The Open University, Milton Keynes, United Kingdom; 2 Faculty of Life Sciences, The University of Manchester, Manchester, United Kingdom; 3 Department of Animal and Plant Sciences, University of Sheffield, Sheffield, United Kingdom; 4 Department of Mathematics and Statistics, The Open University, Milton Keynes, United Kingdom; 5 Department of Earth and Environmental Sciences, The Open University, Milton Keynes, United Kingdom; 6 Department of Community Ecology, Helmholtz Centre for Environmental Research – UFZ, Halle, Germany; 7 Institute of Plant Sciences, University of Bern, Bern, Switzerland; 8 Section of Conservation Biology, Department of Environmental Sciences, University of Basel, Basel, Switzerland; 9 Netherlands Centre for Biodiversity Naturalis, Leiden, The Netherlands; 10 Département Ecologie et Gestion de la Biodiversité, Muséum National d'Histoire Naturelle, Paris, France; 11 Department of Invertebrate Zoology, Natural History Museum of Vienna, Vienna, Austria; 12 Department of Biology and Evolution, University of Ferrara, Ferrara, Italy; 13 Ciência Viva - National Agency for Scientific and Technological Culture, IBMC - Instituto de Biologia Molecular e Celular, Universidade do Porto, Porto, Portugal; 14 Museum of Natural History, Wrocław University, Wrocław, Poland; 15 Institute of Biology, Pomeranian University, Słupsk, Poland; 16 Latvia State Institute of Fruit-Growing, Dobele, Latvia; 17 Waterford Institute of Technology, Waterford, Ireland; 18 Department of Gene Technology, Tallinn University of Technology, Tallinn, Estonia; 19 Department of Biological Sciences, Alberta Biodiversity Monitoring Institute, University of Alberta, Edmonton, Canada; 20 Department of Zoology, Hungarian Natural History Museum, Budapest, Hungary; 21 Department of Science, INS Sant Quirze, Sant Quirze del Vallès, Spain; Smithsonian's National Zoological Park, United States of America

## Abstract

Organisms provide some of the most sensitive indicators of climate change and evolutionary responses are becoming apparent in species with short generation times. Large datasets on genetic polymorphism that can provide an historical benchmark against which to test for recent evolutionary responses are very rare, but an exception is found in the brown-lipped banded snail (*Cepaea nemoralis*). This species is sensitive to its thermal environment and exhibits several polymorphisms of shell colour and banding pattern affecting shell albedo in the majority of populations within its native range in Europe. We tested for evolutionary changes in shell albedo that might have been driven by the warming of the climate in Europe over the last half century by compiling an historical dataset for 6,515 native populations of *C. nemoralis* and comparing this with new data on nearly 3,000 populations. The new data were sampled mainly in 2009 through the Evolution MegaLab, a citizen science project that engaged thousands of volunteers in 15 countries throughout Europe in the biggest such exercise ever undertaken. A known geographic cline in the frequency of the colour phenotype with the highest albedo (yellow) was shown to have persisted and a difference in colour frequency between woodland and more open habitats was confirmed, but there was no general increase in the frequency of yellow shells. This may have been because snails adapted to a warming climate through behavioural thermoregulation. By contrast, we detected an unexpected decrease in the frequency of Unbanded shells and an increase in the Mid-banded morph. Neither of these evolutionary changes appears to be a direct response to climate change, indicating that the influence of other selective agents, possibly related to changing predation pressure and habitat change with effects on micro-climate.

## Introduction

**Figure 1:**
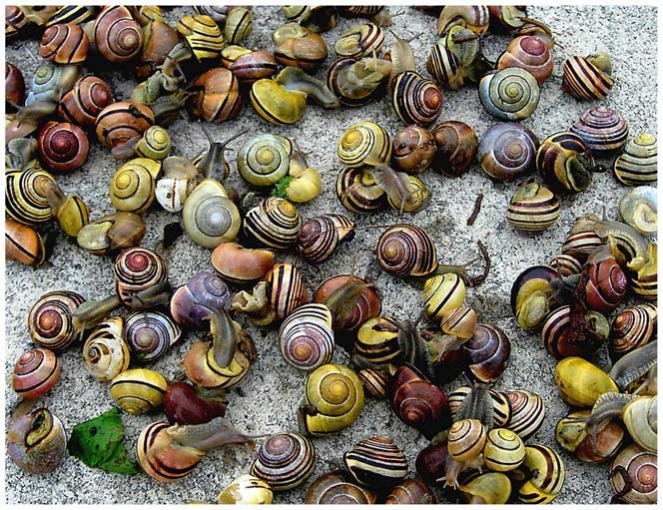
A collection of snails from a polymorphic population of *Cepaea nemoralis* in Poland. This illustrates the variety of shell colours (Yellow, Pink, Brown) and banding (0, 1, 5) typically found. Photograph by Robert Cameron.

Organisms provide some of the most sensitive indicators of climate change [1] and evolutionary responses are becoming apparent in species with short generation times [2], [3]. Pre-existing genetic polymorphism in populations provides the raw material for evolutionary adaptation to climate change [4], [5], [6], [7] and historical gene frequencies are also a benchmark against which evolutionary response can be measured [8], [9]. However, genetic data that cover the full geographic range of a species before warming and other global changes such as biodiversity loss occurred are scarce. An exception is the banded snail *Cepaea nemoralis* that exhibits several polymorphisms affecting shell colour and banding pattern in the majority of populations within its native range in Europe [10] (Fig. 1). Decades of research concentrated between 1930–1980 recorded local and continental-scale associations between particular phenotypes, habitat and climate.

When exposed to sunlight, the colour of a snail's shell influences the temperature experienced by the animal within [11]. Hence in *Cepaea nemoralis* and other polymorphic snails, shell colour and the presence or absence of dark bands that absorb solar radiation influence behavioural thermoregulation [12], [13], [14] and the biochemistry of response to environmental temperature differs between morphs [15], [16]. Within populations, behavioural thermoregulation produces correlations between morph and microhabitat [17], [18] and thermal gradients within a habitat can therefore contribute to the maintenance of shell polymorphism in snails [19], [20], [21], [22]. Cepaea species are subject to predation, especially by birds, so that in any given habitat inconspicuous morphs may have an advantage over conspicuous ones [23]. Visual selection and selection based on thermal effects of colour may be confounded [24]. At the larger, geographic scale, morph frequencies in *Cepaea nemoralis* exhibit a cline, with the frequency of Yellow (the shell colour with the highest albedo) declining northwards in Europe [10].

The average land temperature in Europe increased by 1.3°C during the twentieth century, with a particularly steep rise between 1990–2009 [25]. We proposed that this increase in environmental temperature might have produced selection in favour of shell morphs with a higher albedo (Yellow, Unbanded or 1-banded) at the expense of morphs with a lower one (dark pink or Brown, Many-banded shells). We tested this hypothesis using a new compilation of the historical data on morph frequencies in *C. nemoralis* and a large, new continent-wide dataset on *Cepaea* polymorphism that we collected in 2009. The new data were collected through a citizen science project, the Evolution MegaLab [26], that engaged thousands of volunteers in 15 countries throughout Europe in the biggest such exercise ever undertaken.

## Methods

### *Cepaea nemoralis*

*C. nemoralis* is among the largest and because of its polymorphism and bright colours one of the most easily identified snails in Western Europe. It is a very common and widespread species, occupying a very wide range of habitats from dunes along the coast to woodlands with full canopy cover. No doubt aided by human transport, it is a good colonizer, and is often found in gardens, parks and abandoned land in cities. It is comparatively slow-growing, usually taking three years to develop from egg to breeding adult. It feeds mainly on dead or senescent plants. Like most Pulmonate land snails, it is hermaphrodite and must mate to produce fertile eggs. Mating tends to be concentrated in late spring and early summer, though it can continue through the autumn. The snails often store the sperm they receive from their partner for some time, and individual broods can have mixed paternity. In winter, the snails may hibernate, but can be active in warm spells.

### Data

A scoring scheme was devised for snail morphs and for the different kinds of habitats in which they are found that would enable us to compare data collected by professional biologists during the twentieth century with data that could be reliably collected by volunteers. The ground colour of the shell is controlled at a single locus (‘C’) and displays three main phenotypes: Brown, Pink or Yellow, in order of decreasing dominance [27]. Pink and Brown tend to be difficult for amateurs to tell apart, but Yellow is easily distinguished. We asked participants to record all three colours, but here have analyzed only the frequency of the homozygous recessive Yellow morph relative to other shell colours. Presence of banding is controlled by a locus (‘B’) at which a dominant allele suppresses banding. This locus is linked to the ground colour locus C and produces phenotypes that are either Unbanded or banded (homozygous recessive). The maximum number of bands is five. A number of loci control the number and appearance of bands by modifying the expression of B. We scored the effect of the locus (‘U’), unlinked to C and B. The dominant allele at this locus suppresses bands 1,2, 4 and 5, producing a single, Mid-banded phenotype, the homozygous recessive showing all five bands. Accurate scoring of other banding variants is hard for amateurs; hence, we scored banding phenotypes as Unbanded, Mid-banded or Many-banded, and further variation in this character is excluded from our analyses.

We scored the habitats in which all *C. nemoralis* populations were sampled in the four categories: woodland, hedgerow (lines of shrubs & trees at field & other boundaries), grassland or sand dune. These habitats are generally distinct enough not to be confused with each other by amateurs and can also be distinguished in aerial photographs, so that quality control checks could be performed at sample locations using Google Earth.

Locations of published samples were usually given in the source as map references or place names which we translated into latitude and longitude. In the Evolution MegaLab (http://www.evolutionmegalab.org) the latitude and longitude of every sample was recorded by asking users to pinpoint its location using an embedded Google map.

Data on the frequencies of shell morphs, habitats and locations recorded in the twentieth century, and in just a few cases earlier, were captured from the published literature, from theses and from public and personal archives (R.A.D. Cameron, J.S. Jones and J.J.D. Greenwood). We refer to this dataset as the historical record and it contained data on 6,515 populations of *C. nemoralis* sampled throughout Europe. The bulk (89%) of the samples in the historical dataset were collected between 1950 and 1990. A modern dataset was compiled from professional and amateur samples made between 2000 and 2009, the majority being collected in 2009 by volunteers participating in the Evolution MegaLab. The modern dataset comprised samples from 2,990 populations of *C. nemoralis* containing at least 10 individuals. Over half a million snails were sampled in the entire dataset.

The Evolution MegaLab operated through a website specially designed for this study. The website displayed a map of all the historical data so that users could re-sample the locations of past records, although most new records were not made at the locations of previous ones. The entire website and supporting training materials were produced in 14 language versions and were used by collaborators in 15 European countries (see author list) to solicit data from the general public. Colour identification guides and videos instructed participants on how to sample, how to identify *Cepaea*, how to distinguish *C. nemoralis* from *C. hortensis* (also recorded, but data not reported here), how to distinguish morphs from each other and how to record the data. A quiz was used to train participants in recognizing the correct snail species and their different morphs and to simultaneously test and record their ability to make the correct choices.

We instructed participants to record only mature snails with a lip to the shell because its colour distinguishes *C. nemoralis* (dark brown lip) from *C. hortensis* (white lip). The quiz results indicated that users were readily able to distinguish mature *C. nemoralis* from *C. hortensis*, but that juvenile *C. nemoralis* could be mistaken for mature *C. hortensis*. For this reason, we have confined the current analysis to *C. nemoralis*. We removed obviously duplicate samples and samples where an excess of morphs known to be rare or absent in the species (Brown Mid-banded and Brown Many-banded) suggested that some other species such as *Cornu aspersum* or *Arianta arbustorum* had been recorded in error. Only 29 samples, or 1% of the original dataset, needed to be removed for this reason. Further details of how the Evolution MegaLab was planned and operated are given elsewhere [28].

Mean surface climate data per cell on a 0.25 degree grid, as described by Haylock et al [29], were obtained from the E-OBS dataset from the EU-FP6 project ENSEMBLES (http://ensembles-eu.metoffice.com) via the Climate Explorer website (http://climexp.knmi.nl). The altitude of each sample location was derived from a 30 arc second digital elevation model (GTOPO30 http://eros.usgs.gov/#/Find_Data/Products_and_Data_Available/gtopo30_info) i.e. at much higher resolution than the climate data. This altitude information was used to adjust the temperature value at each sample location relative to the average altitude of the climate data grid at that point, based on a lapse rate of 6 degrees K per 1000 m altitude. This adjustment had little effect on the temperature values in the lowlands but changed some of the values by several degrees in mountainous regions.

### Statistical analysis

The data were analysed by generalized additive models [30] using the gam function from the mgcv package (version 1.6–2) [31] running in the R statistical environment (http://cran.r-project.org). Separate models were run for the frequency of each of three phenotypes, but each model had the same structure and was run on the historical and modern datasets combined. The three dependent variables were: frequency of Yellow, frequency of Mid-banded as a percentage of banded shells, and frequency of Unbanded. The distributional model used was “quasibinomial” with a logistic link function; that is (for Yellow), the number of Yellow shells was assumed to have a binomial distribution with sample size n, the total number of snails observed in the population, and with probability p such that log(p/1-p) is given by a smooth additive function of the independent variables. To allow for overdispersion, the dispersion parameter was estimated from the data rather than using the fixed value of 1 that arise from the binomial distribution, and approximate t and F tests were used to compare models.

A list of independent variables used is given in Table 1. The Temp term modeled the effect of temperature as a joint smooth function of both the January minimum and July maximum temperatures, while the Location term modeled the effect of geographic location as a joint smooth function of latitude and longitude, independently within the modern and the historical samples. This allowed not only for geographic variation in phenotype frequencies that was unrelated to temperature, altitude or habitat, but also for the different geographic distributions of sampling points in the historical and modern datasets. The two-category factor ModHist was also included as a separate term to account for extraneous differences between the historical and modern data sets.

**Table 1:** Definitions of independent variables used in the statistical analysis.

ModHist	A 2-category factor that distinguishes samples collected post-2000 (mainly in the EML in 2009) from those collected in the 20th Cent and earlier.
Year	Range 1909–2009. Fitting Year as well as ModHist enables us to distinguish time effects from other cause of differences between the modern and historical samples.
Habitat	4-category factor representing the habitats (woodland, hedgerow, grassland, sand dune) recognized in the EML. Note that the results shown in Table 2 represent the value of 3 of the habitats with respect to Woods.
Alt	Altitude of the sample location in m above sea level, derived from a digital elevation model, (GTOPO30) with a horizontal grid spacing of 30 arc seconds (approximately 1 kilometre)
JanPrecip	Average January precipitation mm/day
JulyPrecip	Average July precipitation mm/day
Temp	Temp modeled the effect of temperature as an interaction between January minimum and July maximum temperatures.
Location	A term including an interaction between latitude and longitude, fitted independently within the modern and the historical samples. This was used to remove the deviance due to samples being made in different places in the historic and modern periods.

For each phenotype, the model fitted to the probability (p) of the phenotype was: log(p/(1−p)) = ModHist+Year+Habitat+s(Altitude)+s(JanPrecip)+s(JulyPrecip)+s(Temp)+s(Location). Terms with an ‘s’ prefix were smoothed functions rather than linear, to improve fit. In a model with a spatial aspect like this, there is a risk that standard fitting processes will be compromised by spatial autocorrelation. Preliminary analyses of spatial correlograms and variograms indicated that in fact the degree of spatial autocorrelation in the phenotype frequencies was relatively low (and practically nonexistent at scales above 20 km), and similar analysis of the residuals from the fitted model showed negligible levels of spatial correlation in the residuals. Some additional models with fewer terms or with data restricted to the historical period were also run to test for the dependence of overall model results on the presence of particular variables such as temperature and to check for artifacts related to ModHist.

A smaller-scale, more direct analysis was performed on 554 pairs of samples where records were made in the same habitat type within 20 km of each other in both the historical and modern sampling periods. We broke the pairs down into the four habitat types and compared historical and modern samples using paired-sample t-tests on arcsine transformed frequencies.

We investigated the potential relationship between the increase in the frequency of Mid-banded and temperature by testing the hypothesis that Mid-banded would have increased most in places that had warmed the most between the historical and modern periods. There were insufficient samples made at the same locations in the two periods for this test to be performed by direct comparison of the historical and modern data. We therefore used the model to predict how much change in the frequency of Mid-banded had taken place over the 50 years from 1950–2000 at each of 2,827 locations sampled in the Evolution MegaLab. (Temperature change could not be computed for 163 of the 2,990 locations sampled.) These model predictions were used as surrogates for actual frequency change and correlated with the actual recorded changes in temperature at each location. Note that the predictions were compared with temperature change, and not temperature, which was a term in the model. This analysis is therefore going beyond the original modelling, to investigate a possible reason for the model looking as it did, and is not validating the model itself.

## Results

### Evolutionary change in phenotype frequencies

Evolutionary change in phenotype frequencies detected in the gam analysis is indicated by significant Year terms in Table 2. Yellow decreased over time, but the magnitude of the effect was very small (α = −0.002) and its significance was marginal (P = 0.036) considering the size of the dataset (n = 9,505). Removing temperature from the model made the change even smaller and non-significant (α = −0.001, P = 0.272). In contrast, banding patterns did change significantly with time. The frequency of Unbanded decreased over time and the frequency of Mid-banded as a proportion of the banded fraction increased (Table 2). Neither of the changes in banding was much affected by the removal of temperature from the full model (Unbanded α = −0.010, P<0.001; Mid-banded α = 0.008, P<0.001). The increase in Mid-banded was also found in analyses confined to the historical dataset (α = 0.0166, t = 12.891, P<0.001) and of the combined dataset without the sand dune samples (where Mid-banded was significantly rarer, Table 2) (α = 0.0119, t = 9.185, P<0.001).

**Table 2:** Summary of the results of General Additive Modeling of shell polymorphism in *Cepaea nemoralis* in the combined dataset of historical and 2009 samples collected throughout Europe.

Phenotype	n	Dev%	ModHist	Year	Habitat				Alt	JanPrecip	JulyPrecip	Temp	Location	
					Hedge	Grass	Dune	Overall					Hist	Mod
Yellow	9505	32.8%												
α			0.379	−0.002	0.509	0.649	1.350							
t/F			2.560	−2.097	14.041	15.428	20.415	†	0.139	10.200	14.697	12.367	32.939	9.142
P			0.011	0.036	<0.001	<0.001	<0.001	†	0.71	<0.001	<0.001	<0.001	<0.001	<0.001
Mid-banded	9325	37.2%												
α			−0.096	0.009	−0.075	−0.039	−0.798		5.356					
t/F			−0.632	7.114	−1.781	−0.820	−9.776	46.426	4.056	26.507	6.639	15.190	36.853	17.182
P			0.527	<0.001	0.075	0.413	<0.001	<0.001	<0.001	<0.001	<0.001	<0.001	<0.001	<0.001
Unbanded	9505	28.9%												
α			0.146	−0.009	−0.119	−0.010	−1.159		7.442					
t/F			0.972	−7.938	−2.886	−0.214	−11.836	50.956	9.769	16.023	11.536	8.347	24.313	5.436
P			0.331	<0.001	0.004	0.830	<0.001	<0.001	<0.001	<0.001	<0.001	<0.001	<0.001	<0.001

Statistics shown are coefficients (α) and t-values for unsmoothed terms and F-values for smoothed terms. P-values are approximate. n = sample size (number of populations), Dev% is the percent deviance accounted for by the model. Mid-banded frequency is calculated as a percentage of banded. Coefficient values are not given for the terms entered as smooth functions, because such function cannot be described by a single coefficient.

† In this case the model omitting Habitat as an independent variable fits slightly better in terms of deviance than the model including Habitat, so no test statistic or p value can be calculated.

The results of the paired-sample analysis are given in Table 3. A significant increase in the frequency of Yellow occurred only in dunes where there was also a marginally significant increase in Mid-banded. An increase in Unbanded was detected in hedges, where Mid-banded also increased very significantly.

**Table 3:** Paired sample analysis for sites sampled within 20 km of each other in the historical and modern datasets.

Habitat/morph	n	Frequency			t	P
		Historic	Modern	Change		
Woods	90					
Yellow		0.376	0.437	+0.061	1.830	0.071
Unbanded		0.228	0.279	+0.051	1.991	0.050
Mid-banded		0.347	0.368	+0.021	0.269	0.789
Hedge	245					
Yellow		0.583	0.561	−0.023	−1.118	0.265
Unbanded		0.240	0.279	+0.038	2.403	0.017
Mid-banded		0.267	0.348	+0.081	3.708	>0.001
Grass	65					
Yellow		0.619	0.614	−0.005	0.006	0.995
Unbanded		0.263	0.298	+0.034	0.896	0.374
Mid-banded		0.401	0.480	+0.079	1.622	0.110
Dunes	52					
Yellow		0.534	0.672	+0.138	2.875	0.006
Unbanded		0.117	0.133	+0.016	0.745	0.460
Mid-banded		0.182	0.263	+0.081	2.177	0.034

n = number of pairs.

### Effects of altitude, climate and location on phenotype frequencies

**Figure 2:**
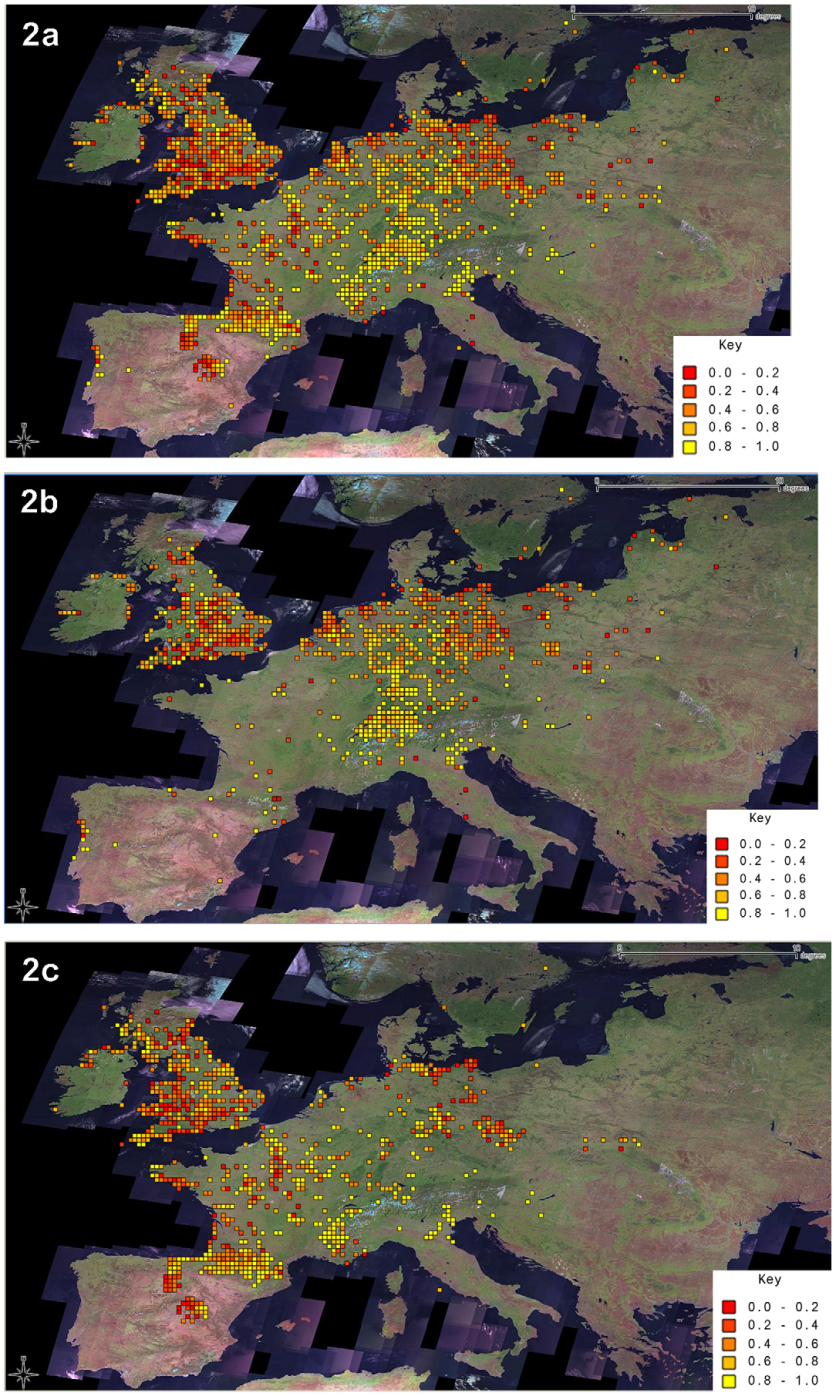
Frequency of Yellow *C. nemoralis* recorded as population averages in quarter degree cells of latitude and longitude. (a) the modern and historic datasets combined (b) the modern dataset and (c) the historical dataset. The Key shows the division of the frequency of the Yellow morph by intervals of 0.2.

The modern dataset displayed the expected cline in the frequency of the Yellow morph (Fig. 2b) also found in the historical dataset (Fig. 2c). Location was highly significant in all models and differences between the location terms in the historic and modern datasets were also significant (Table 2). Mid-banded and Unbanded both increased with altitude, but Yellow was unaffected by altitude. All phenotype frequencies measured increased with increases in precipitation and temperature (Table 2). The correlation between predicted changes in the frequency of Mid-banded and geographic variation in temperature change between 1950–2000 was negative and highly significant (r = −0.108, d.f. = 2825, P<0.001).

### Effects of habitat on phenotype frequencies

**Figure 3:**
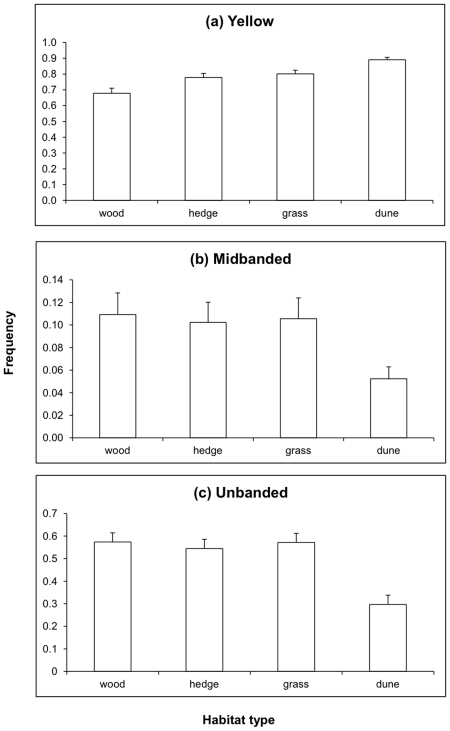
Frequency of morphs in four habitat types, as predicted from the models in **Table 2**. Standard errors are shown. (a) Yellow, all differences between habitats are significant P<0.001; (b) Mid-banded, differences between dune habitat and all others are significant P<0.001. Other differences are not significant; (c) Unbanded. All differences are significant except for that between grassland and woodland. (P<0.001 for all comparisons involving dune, P = 0.023 for woodland v hedge, P = 0.027 for hedge v grassland). All P values in these comparisons were corrected for multiple testing using Bonferroni.

The frequency of Yellow was affected by habitat (Table 2), increasing from woodland (the most shaded habitat) to sand dunes (the most exposed). Mid-banded and Unbanded were less frequent in sand dunes than in the other habitats (Fig. 3).

## Discussion

Our dataset on shell polymorphism in *Cepaea nemoralis* is unique among genetic studies of non-human organisms for its combination of wide geographic scale, deep historical scope and the large number of individuals scored. By combining habitat, climate, and geographic variables in spatially-informed models, we have obtained a comprehensive picture of evolutionary change over the recent period of anthropogenic global change. The time elapsed between the heavy sampling of *C. nemoralis* in the 1960s and '70s and our major sampling effort in the Evolution MegaLab is 40–50 years, or about 15–20 *Cepaea* generations. We hypothesized that the increase in environmental temperature that occurred towards the end of the twentieth century would have favoured shell morphs with a higher albedo. Although we did detect evolutionary changes, those found in our most comprehensive analyses were either not in the direction predicted or they were uncorrelated with local temperature change and so did not support our hypothesis.

The cline in the frequency of the Yellow morph known from the historical period [10] persisted into the 21st Century (Fig. 2). In the full statistical model, the Yellow morph decreased slightly in frequency over time, but only at a marginal level of significance (P = 0.036, Table 2). If a warming climate had driven an increase in the frequency of Yellow, a correlation between the two variables in the full model could hide an increase in Yellow. If this was the case, then removing temperature from the model ought to reveal any increase in Yellow that had actually occurred. However, when we dropped temperature from the model, Yellow was found not to have changed at all. The paired sample analysis, which utilized a small subset of our data comprising only about 10% of all samples, found an increase in the frequency of Yellow in dunes, but not in the other three habitat types (Table 3).

This difference between sand dunes and the other habitats which provide more vegetation cover may provide a clue as to why the expected increase in Yellow was not general. Behavioural and physiological adaptation to temperature can buffer evolutionary responses to a warming climate [32]. Behavioural thermoregulation occurs in *Cepaea* and varies between morphs [12], but snails require shaded refuges into which they can move if this behaviour is to be effective. Such refuges are more available in woods, hedges and grassland than in sand dunes, and this may explain why only in sand dunes was there an increase in the frequency of Yellow morphs. It would also explain the increases in frequency of Yellow with decreasing shadedness of habitat in the order wood<hedge<grass<sand dune (Fig. 3 & Table 2).

Two evolutionary changes in banding, controlled by two different, unlinked loci, were detected. The frequency of Unbanded decreased over time in the complete dataset (Table 2), though this change was absent or reversed in the paired sample analysis (Table 3). The frequency of Mid-banded (among banded) increased in the dataset as a whole (Table 2) and among the 245 paired samples taken from hedges (Table 3). The magnitude of these frequency changes is better estimated from the models than from changes in raw average frequencies since the models allow for different geographic distributions of sampling points in the historical and modern data sets that could bias raw averages. The models estimated a 10% decrease in Unbanded and a 5% increase in Mid-banded had taken place. The 10% decrease in Unbanded is a surprisingly large change to observe in only 15–20 generations and may be an over-estimate. The 5% increase in Mid-banded is a more robust estimate as change of the same order was also found in the paired-sample analysis of hedge populations (Table 3).

Bands are darker than the ground colour of all shell phenotypes and therefore a general decrease in Unbanded (and a corresponding increase in banding) does not support the climate warming hypothesis because this predicts that phenotypes with lower albedo ought to decrease. The increase of Mid-banded, which took place at the expense of morphs with more bands, was in the direction expected from the climate hypothesis and so we applied an additional test. If the climate hypothesis is correct, then the increase in Mid-banded should have been greatest where the temperature increased the most. Our test for this was based upon model predictions and showed that the change in Mid-banded frequency did correlate with local changes in temperature, but that the correlation was negative rather than positive and therefore in the wrong direction to support the hypothesis. The climate hypothesis is therefore not supported and we must look to another explanation for why the frequency of Mid-banded has increased.

Turning to the association between morph and habitat, a strong inference from earlier work was that open habitats have higher frequencies of Yellow and banding than woodland. This observation was made in a mature agricultural region of England [23], where Mid-banded also declined from woods to open areas. A review of results from several studies [33] confirmed the colour and banding associations, though the Mid-banded effect was not consistent. In the present analysis (Table 2) the frequency of Yellow is undoubtedly associated with habitat, increasing from woodland (the most shaded) to sand dunes (the most exposed) (Fig. 3), but with respect to the banding patterns the result with the full data set differs from that recorded earlier for particular locations. Unbanded is lower on sand dunes than in woods and marginally so in hedgerow habitats (Fig. 3). No difference in Unbanded was detected between woodland and grass. Mid-banded was less frequent in dunes than in the other habitats (Fig. 3).

When Cain and Sheppard [23] made their original observations they emphasized that woodland samples were darker and more uniform in phenotype than those from open habitats and concluded that this association was due to visual predation tending to increase the match of the samples to their backgrounds. The main predator is the song thrush *Turdus philomelos*. However, such an association could also be consistent with a response to microclimate [24], [34]. In the modern data the association depends almost entirely on Yellow frequency. The habitat effect may therefore be related to the albedo effect of Yellow that has been revealed on the larger scale, rather than to predation. It should be noted, however, that many ecological changes have occurred between the times of the two surveys, making it difficult to draw general conclusions. Fluctuations in abundance of thrushes must have affected their capacity to exert selection on the snails. Some long-term studies of *Cepaea* have recorded vegetational changes coinciding with massive declines in rabbit densities due to myxomatosis [35], [36] which must have modified microclimates. Increases in the area of woodland and changes in woodland management have also occurred in Europe over the period of study and these may have been a cause of the increase in Mid-banded due to its lower albedo [24].

By analyzing data on *Cepaea nemoralis* polymorphism and its evolution on a continent-wide scale we have traded the precision of the numerous previous, mainly local studies of this model organism for generality (Fig. 2). In the past, such generalities have been hard to come by in the ecological genetics of *Cepaea* [10], with much evidence that the forces shaping the evolution of *Cepaea* polymorphism can be intensely local and vary from place-to-place. We attempted to deal with some of this spatial variation by including a Location term in our models. This term also allowed for differences in the geographical distribution of samples between the historical and modern datasets and was significant in the models for all three traits (Table 2). This result indicates that we successfully captured geographic variation in phenotype frequencies not accounted for by the other variables (altitude, habitat, climate, year). Some of this variation must have included effects such as drift, and the well-known ‘area effects’ found in *Cepaea* [37] that are probably a consequence of founder effects [38].

The comprehensive data collected by volunteers in the Evolution MegaLab allowed us to apply a powerful test of an evolutionary hypothesis linking climate change with polymorphism in the banded snail. That hypothesis was clearly rejected, but to our surprise, the study also produced another unequivocal result, showing that banded morphs in general and Mid-banded morphs in particular increased. These evolutionary trends do not appear to be related to climate warming and may be related to changing predation pressure by birds. Thanks to the enthusiasm of the general public for bird watching, this hypothesis provides another opportunity for investigation using the power of citizen science.
